# A Genetic Algorithm to Combine Deep Features for the Aesthetic Assessment of Images Containing Faces

**DOI:** 10.3390/s21041307

**Published:** 2021-02-12

**Authors:** Luigi Celona, Raimondo Schettini

**Affiliations:** Department of Informatics, Systems and Communication, University of Milano-Bicocca, viale Sarca, 336, 20126 Milano, Italy; raimondo.schettini@unimib.it

**Keywords:** image aesthetics, faces, convolutional neural networks, genetic algorithms

## Abstract

The automatic assessment of the aesthetic quality of a photo is a challenging and extensively studied problem. Most of the existing works focus on the aesthetic quality assessment of photos regardless of the depicted subject and mainly use features extracted from the entire image. It has been observed that the performance of generic content aesthetic assessment methods significantly decreases when it comes to images depicting faces. This paper introduces a method for evaluating the aesthetic quality of images with faces by encoding both the properties of the entire image and specific aspects of the face. Three different convolutional neural networks are exploited to encode information regarding perceptual quality, global image aesthetics, and facial attributes; then, a model is trained to combine these features to explicitly predict the aesthetics of images containing faces. Experimental results show that our approach outperforms existing methods for both binary, i.e., low/high, and continuous aesthetic score prediction on four different image databases in the state-of-the-art.

## 1. Introduction

Image aesthetic quality assessment (IAQA) is an important visual task, which represents an important criterion for visual content curation and lays the foundation in many multimedia applications such as image retrieval [1,2], photo enhancement [3], and image cropping and photo album creation [4,5,6]. The goal of IAQA is to design algorithms that automatically predict image aesthetic quality. This is a challenging task due to its fuzzy definition and its highly subjective nature. The aesthetic score of images relies on several undetermined factors, such as composition, color distribution, and technical quality. Many approaches for the aesthetic assessment of images with generic content are present in the literature [6,7,8]. However, psychology research [9] shows that certain kinds of content are more attractive than others. Professional photographers adopt different photographic techniques and have various aesthetic criteria in mind when taking different types of photos; therefore, it is reasonable to design features specialized in modeling aesthetic quality for different kinds of photos (e.g., [10,11,12]).

**Figure 1:**
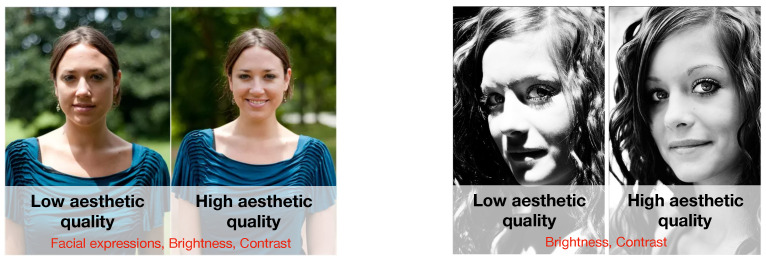
Face aesthetics represents the attractiveness of the photo shot. This takes into account aspects such as: facial expressions, brightness, contrast, etc.

In this paper, we focus on the aesthetic quality assessment of images containing human faces. The reasons are twofold: (i) a large percentage of images on social media sites and media content repositories contains faces and self-portraits, or “selfies” [13,14]; (ii) the performance of generic content aesthetic assessment methods [7] drops considerably when dealing with these types of images. The automatic estimation of the overall aesthetics of images containing faces is fundamental for a wide range of applications, for example to discriminate professional and amateur portraits on sharing platforms [15], to choose the most aesthetically pleasing picture for sharing on social media [16], to guide the capturing process on smart cameras [17], or to handle the automatic creation of photo albums [1]. The prediction of the overall aesthetics of an image containing faces is the result of the combination of several features encoding relevant information about the global image aesthetics adapted to facial pictures, as well as information related to facial expressions and high-level attributes (e.g., smile, age, gender, hair style). It should be clear that although facial beauty and face aesthetics are two related concepts, the first reflects the attractiveness of the subject’s face, while the second represents the attractiveness of the photo containing the subject’s face (see, for example, Figure 1).

Previously proposed methods for the aesthetic quality assessment of images containing faces can be grouped into those that treat the problem as a categorization into images with low or high aesthetic quality [18,19,20] and those that instead estimate a continuous score of aesthetic quality [1,17,19].

Males et al. [18] exploited a support vector machine for aesthetic quality categorization trained on the combination of global (e.g., contrast and hue distribution of the whole image) and local features (e.g., sharpness and blown-out highlights only of facial region). Their experiments were carried out on a set of photos collected from Flickr and manually labeled by five people as being aesthetically appealing or not. In [20], a compositional based augmentation scheme was used to train a deep convolutional neural network (DCNN) on a portrait subset of the AVA dataset for binary aesthetic classification. Li et al. [21] evaluated the performance of several categories of features related to aesthetics such as pose, face locations, and photo composition on their own dataset of photos with faces. Lienhard et al. [19,22] proposed a new database, called Human Faces Score (HFS), and developed a method based on the selection of low-level features extracted from several regions for both aesthetic quality categorization of portrait images (i.e., low or high) and continuous aesthetic score prediction. Recently, many works have proposed intelligent capture methods for taking good selfies based on hand-crafted features and face pose analysis [17,23].

In this paper, we propose a method for the aesthetic assessment of images containing faces. It involves the use of three convolutional neural networks (CNNs) to encode information regarding perceptual quality, global image aesthetics, and facial attributes. A mixed-coded genetic algorithm (GA) is trained to combine these features to explicitly predict the aesthetics of images containing face. The mixed-GA is built to simultaneously address: (i) the selection of relevant features and (ii) the optimization of the weights characterizing the linear model, which maps features to an aesthetic prediction. As far as we know, this is the only approach that, for estimating the aesthetic quality of images containing faces, takes into account the properties of the entire image, as well as aspects specific to the face such as demographic attributes (gender, age, and ethnicity), mood (facial expressions), and visual attributes (e.g., hair style, clothing, face shape).

The idea underlying this method was presented in [24]. In this paper, we revise this idea, and in particular, we perform a deeper investigation concerning the fitness functions to be used for the optimization of the genetic algorithm. We also exploit a richer set of evaluation metrics to more comprehensively assess the aesthetics models. Moreover, a new set of experiments assessing the generalization ability of the best method is carried out.

The rest of the article is organized as follows: Section 2 details the proposed method; in Section 3, we present the experimental protocol and the considered metrics; Section 4 reports the results and the analysis of the performance achieved; and conclusions and comments are made in Section 5.

## 2. Facial Image Aesthetic Estimation

**Figure 2:**
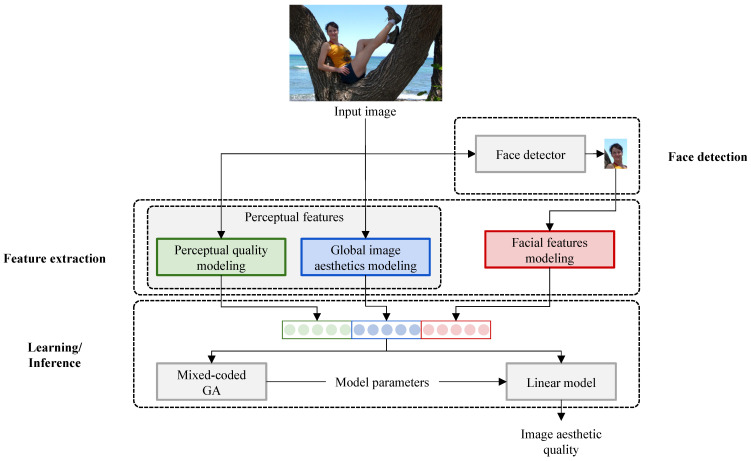
Overview of the proposed method. Given an image containing faces, the largest face is detected and cropped. Perceptual features are extracted from the whole image, while facial features are computed on the crop of the face. A mixed-coded genetic algorithm (GA) is used for estimating the parameters of a linear model, which predicts the image’s aesthetic quality.

In this section, we describe the proposed method for the aesthetic quality assessment of images with faces. The proposed method is depicted in Figure 2: given a photo, first, the largest face is detected, then features are extracted from the whole image and the face region, and finally, the trained model is applied for the aesthetic quality estimation of the photo.

### 2.1. Face Detection

Faces are detected in the input image using the RetinaFace detector [25] with the ResNet-50 backbone. RetinaFace is a robust single-stage face detector capable of simultaneously locating the face region, predicting the coordinates of five landmarks for the eyes, nose, and mouth, and estimating the pixel-wise 3D shape face information. The size of the detected bounding box is increased by 10% to also include a portion of the shoulders; the facial region is then cropped from the entire image, and no alignment is adopted. In the presence of multiple faces within an image, the largest one is considered.

### 2.2. Feature Extraction

The aesthetic quality of photos with generic content, as well as the aesthetics of photos with faces depend on several perceptual properties. Furthermore, face attributes provide fundamental information for the aesthetic evaluation of this specific category of photos. In this paper, we use state-of-the-art CNNs for encoding both perceptual image-related and face properties. As highlighted in many previous works, aesthetic quality is strongly influenced by several dimensions such as composition, colorfulness, spatial organization, emphasis, and depth. We consider two pre-trained CNNs for image quality assessment and generic content aesthetic assessment, proposed in the authors’ previous works, in order to encode such information about the whole image (face and background).

**Figure 3:**
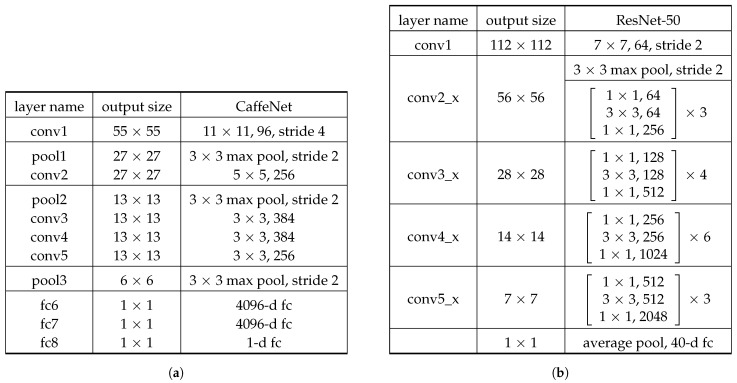
The CNN architectures of the feature extractors. (**a**) CaffeNet architecture used for DeepIA [7] and DeepBIQ [26]. (**b**) ResNet-50 architecture used in Alignment-Free Facial Attribute Classification Technique (AFFACT) [29].

**Figure 4:**
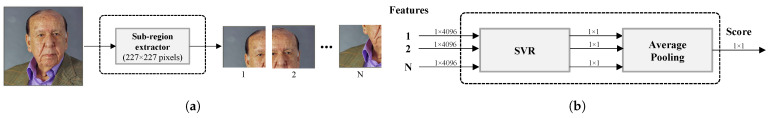
The main components of the DeepBIQ model. (**a**) The sub-region extractor, which divides an image of variable resolution into a grid of overlapping sub-regions of size 227 × 227 pixels. (**b**) The feature vectors extracted from the CNN for each sub-region are fed into the SVR, which predicts a quality score for each of them. The quality score for the entire image is calculated by average pooling the predicted scores on all the sub-regions of the original image.

For encoding perceptual quality metrics such as noise, exposure, quality, JPEG quality, and sharpness, we use the DeepBIQ model [26] (IQ for short), which is one of the state-of-the-art methods for blind image quality assessment [27]. It involves a feature extractor, consisting of CaffeNet (see the architecture in Figure 3a) trained to classify images into five image quality grades, followed by a support vector regressor (SVR) to map the feature vector into a quality score. Given an input image with a variable resolution, it is divided into a grid of 227×227 overlapping sub-regions (see Figure 4a). For each sub-region, the CNN then performs all the multi-layered operations, and the corresponding feature vector is obtained by removing the last fully-connected layer. The 4096-dimensional feature vectors of all the sub-regions are fed into the SVR, which predicts a region-level quality score. The quality score for the whole image is computed by average pooling the scores predicted on all the sub-regions of the original image (see Figure 4b). In this work, the feature vectors of each sub-region are averaged to obtain a representation of the whole image. The obtained feature vector has 4096 elements.

To extract features related to global image aesthetic concepts, such as brightness, contrast, and color, we exploit the DeepIA model [7] (IA in short), which is a CNN trained for generic content aesthetic assessment. It consists of a CaffeNet model (see Figure 3a) trained on the AVA dataset [28] to predict the aesthetic score of RGB images of size 227×227. The 4096-dimensional feature vector for this model is extracted by removing the last fully-connected layer.

**Figure 5:**
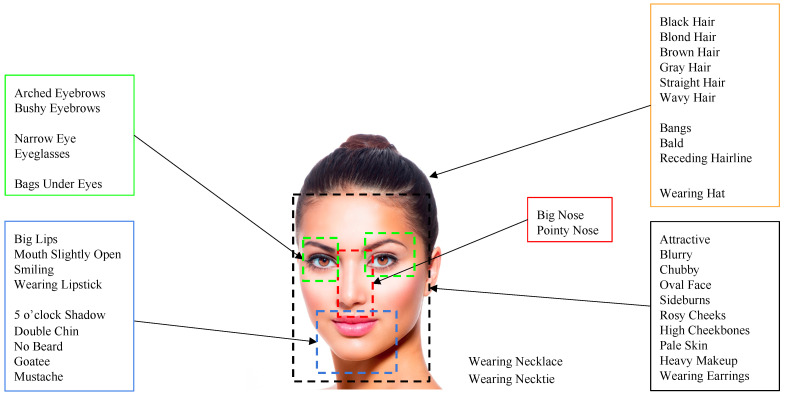
A graphical representation of the 40 attributes used to describe faces.

In photos containing faces, observers mainly focus on face regions. Intuitively, face attributes such as facial expressions, the presence of makeup, or the presence of accessories are closely related to the aesthetics of this specific category of photos. Therefore, we consider a set of features able to accurately describe the face. To this aim, we use the Alignment-Free Facial Attribute Classification Technique (AFFACT) [29] (FA in short), a CNN model (see the architecture in Figure 3b) trained for the estimation of 40 facial attributes (see Figure 5) given an RGB image of 224×224 pixels. The 2048-dimensional vector corresponding to the activations of the fully-connected layer before the classification layer is used as the features.

### 2.3. Feature Fusion and Learning Procedure

As previously stated, the overall aesthetics of an image containing faces results from the combination of several characteristics that encode global image attributes concerning quality and aesthetics and information related to facial attributes [24]. However, we do not know which of these features are relevant, how they are interlaced, or how they change based on how the photo was taken. We let these relationships be learned and modeled directly on the data using the genetic algorithm (GA). To do this, the previously extracted features are fused using linear concatenation, then exploited for the GA based learning procedure. Since the resulting feature vectors have a high number of features (10,240 when all features are chained), some of which might be redundant, the proposed strategy also includes a feature selection step. Feature selection refers to the task of identifying relevant features useful for fitting accurate models. In this work, we propose a GA method to jointly identify a subset of relevant features from the whole feature vector and optimize the parameters of a prediction model. The rationale behind using the GA to handle both problems, i.e., feature selection and learning of prediction model parameters, is that the choice of the prediction model parameters is influenced by the feature subset taken into account and vice versa. Therefore, using a single optimization process allows automatically identifying relevant features and their relationship to the parameters of the inferential method directly from the data.

**Figure 6:**
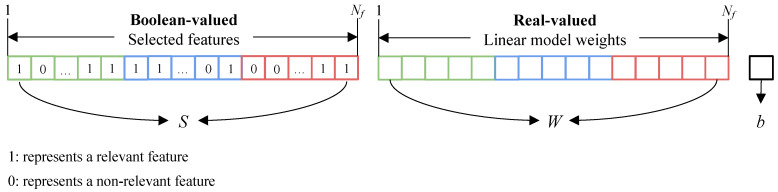
Mixed-coded chromosome used for mapping deep features into an aesthetic prediction. It consists of a Boolean-valued part, *S*, for feature selection, *W* where the elements are encoded by real-valued representation, and *b* is the bias. *S* and *W* have a number of elements corresponding to $N_{f}$, namely the number of features.

The GA is built to solve a mixed integer problem where some variables are restricted to take only integer values. Real-valued variables are the weights (*W*) and the bias (*b*) of the linear model, which maps features to an aesthetic prediction, while the Boolean-valued variables (*S*) discriminate relevant features from the non-relevant ones. Given $j\in [1,N_{f}]$ and $N_{f}$ the total number of features, a chromosome is then represented as θ={S,W,b}, where: $S=\{s_{0},s_{j},\ldots ,s_{N_{f}}\}$ with $s_{j}\Rightarrow \left\{x\in Z:0\leq x\leq 1\right\}$ are binary values coordinating feature selection; $W=\{w_{0},w_{j},\ldots ,w_{N_{f}}\}$ with $w_{j}\in R$ are the weights of the linear model; b∈R is a scalar value indicating the bias term of the linear model that offsets all predictions for a better fit. Figure 6 shows the mixed-coding scheme used for the GA chromosomes.

Given a feature vector *x* and the best fit chromosome θ={S,W,b}, the aesthetic quality is predicted through the following equation:

(1) $$\begin{array}{c} p=\sum_{j=1}^{N_{f}}x_{j}\left(s_{j}w_{j}\right)+b. \end{array}$$

#### Fitness Function

Aesthetic evaluation can be treated as a binary classification problem to discriminate high or low aesthetic quality, or as a regression problem to estimate an aesthetic quality score. For a comprehensive evaluation of the proposed framework, we address both problems, namely two-class categorization and regression. Therefore, for the optimization of the genetic algorithm, we select different fitness functions depending on whether it is a classification or a regression problem.

##### Classification Fitness

The fitness function used for the classification tries to minimize the hinge loss. This loss was primarily developed for Support Vector Machine (SVM) models. It encourages samples to have the correct sign by assigning a larger error when there is a sign difference between the actual and expected class values. It is computed as follows:

(2) $$\begin{array}{c} L_{hinge}=\frac{1}{N}\sum_{i=1}^{N}\sum_{j\neq g_{i}}\max(0,1-\left(p_{j}-p_{g_{i}}\right)), \end{array}$$

where *g* and *p* are the ground-truth and the predicted scores, respectively.

##### Regression Fitness

Three different fitness functions are considered for regression, namely the smooth-L1, the norm-in-norm [30], and the ranking hinge loss. The smooth-L1 loss is widely used for regression tasks because of its robustness to outliers. Given $\left(g_{i},\;p_{i}\right)$, the pair of ground-truth and predicted scores for the *i*-th sample, and *N*, the number of samples, the smooth-L1 loss ($L_{smooth1}$) is computed as:

(3) $$\begin{array}{cc} L_{smooth1}=\frac{1}{N}\sum_{i=1}^{N}d_{i},\;\mathrm{where}\;d_{i} & =\left\{\begin{array}{cc} 0.5{\left(g_{i}-p_{i}\right)}^{2}, & \mathrm{if}\;|g_{i}-p_{i}|<1\\ |g_{i}-p_{i}|-0.5, & \mathrm{otherwise} \end{array}\right. \end{array}$$

**Figure 7:**
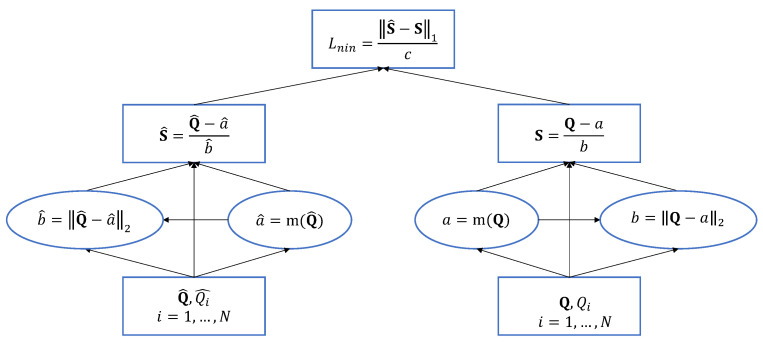
Illustration of the forward pass of the norm-in-norm loss [30]. Q and $\hat{Q}$ are the Mean Opinion Score (MOS) and the predicted quality score vectors, respectively. m(·) denotes the mean function. *c* is a normalization term equal to $2N^{\frac{1}{2}}$.

The recent norm-in-norm loss [30] facilitates faster convergence for training a CNN based (Image Quality Assessment) IQA model and also leads to better prediction performance than the mean absolute error (MAE) and mean squared error (MSE) losses. Its estimation is based on three steps: the computation of statistics, normalization based on the statistics, and loss as the norm of the differences between normalized values. Figure 7 shows each step required to calculate the loss.

The learning-to-rank framework has shown advantages in several computer vision problems over common regression losses [31,32]. Therefore, another fitness function that is used to optimize the genetic algorithm is the ranking hinge loss according to:

(4) $$\begin{array}{c} L_{rank}=\max\left(0,-g\left(p_{i}-p_{j}\right)\right), \end{array}$$

where $p_{i}$ and $p_{j}$ are the predicted scores for two images *i* and *j* and *g* is the label assuming a value of one or −1. If g=1, then it assumes the input *i* should be ranked before the input *j*, and vice versa for g=0.

## 3. Experiments

In this section, the evaluation protocol, the considered databases, and the experimental setup are detailed.

### 3.1. Evaluation Protocol

For the experiments, the same evaluation procedure adopted in [19] was followed. More in detail, for each experiment, ten-fold cross-validation was performed by randomly dividing the dataset into ten disjoint subsets and repeating the experiment ten times, each time selecting a different subset of tests and the remaining nine for training. The division into ten disjoint sets was repeated 10 times to avoid sampling bias.

Classification performance was evaluated in terms of the Good Classification Rate (GCR) and F1 score. The GCR measures the ratio between the number of images correctly classified and the number of test images and is defined as $GCR=CCE\left(0\right)/N_{t}$. The cross-category error (CCE) can be computed as follows:

(5) $$\begin{array}{c} CCE\left(k\right)=\frac{1}{N}\sum_{n=1}^{N}\chi (g_{i}-p_{i}=k), \end{array}$$

where *N* is the number of samples, $g_{i}$ is the ground-truth class, and $p_{i}$ is the predicted class for the *i*-th image. χ(x)=1 if *x* is true, χ(x)=0 otherwise. The F1 score corresponds to:

(6) $$\begin{array}{c} F1=\frac{2\times precision\times recall}{precision+recall}, \end{array}$$

(7) $$\begin{array}{c} precision=\frac{TP}{TP+FP},\;recall=\frac{TP}{TP+FN}, \end{array}$$

where TP is the number of true positives, FP stands for the number of false positives, and FN is the number of false negatives, respectively.

Regression performance was evaluated in terms of Pearson’s Linear Correlation Coefficient (PLCC) and Spearman’s Rank-Order Correlation Coefficient (SROCC). The PLCC measures the linear correlation between the actual and the predicted scores, and it is defined as follows:

(8) $$\begin{array}{c} PLCC=\frac{\sum_{i}^{N}\left(x_{i}-\bar{x}\right)\left(y_{i}-\bar{y}\right)}{\sqrt{\sum_{i}^{N}{\left(x_{i}-\bar{x}\right)}^{2}}\sqrt{\sum_{i}^{N}{\left(y_{i}-\bar{y}\right)}^{2}}}, \end{array}$$

where *N* is the number of samples, $x_{i}$ and $y_{i}$ are the sample points indexed with *i*, and finally, $\bar{x}$ and $\bar{y}$ are the means of each sample distribution. Instead, the SROCC estimates the monotonic relationship between the actual and the predicted scores, and it is calculated as follows:

(9) $$\begin{array}{c} SROCC=1-\frac{6\sum_{i}^{N}d_{i}^{2}}{N(N^{2}-1)}, \end{array}$$

*N* is the number of samples, and $d_{i}=\left(\mathrm{rank}\left(x_{i}\right)-\mathrm{rank}\left(y_{i}\right)\right)$ is the difference between the two ranks of each sample. The average of the considered metrics across the 10 rounds is reported.

### 3.2. Portrait Image Databases

In this section, the publicly available databases for the aesthetic assessment of images with faces are described. The databases consist of images containing people or groups of people gathered from online photo databases or photo sharing websites (e.g., Flickr, DPChallenge). Given that these photos were collected in real scenarios, they present a wide range of subjects, facial appearances, illumination, and imaging conditions.

**Figure 8:**
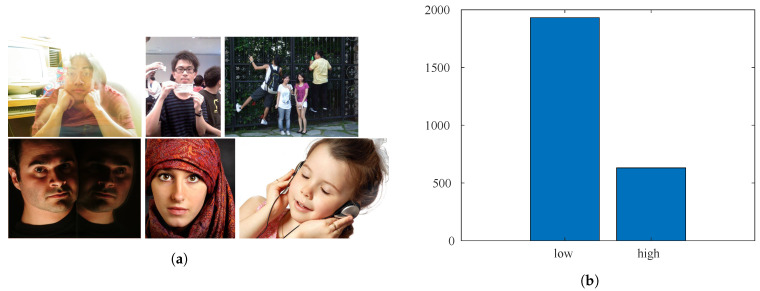
Sample images and number of samples per category for the CUHKPQ database. (**a**) Sample images in the top row were annotated as low aesthetic quality, while samples in the bottom row show high aesthetic quality images. (**b**) Annotation distribution for the low-/high-quality classes.

The CUHKPQ [15] is a manually annotated database for image aesthetics’ categorization (respectively high and low). It consists of 17,673 images organized into seven different categories. In this work, only images belonging to the “human” category are considered. There are 3148 photos of different sizes. The size of the faces instead varies between 180×269 pixels and 1357×900 pixels. Some example images are shown in Figure 8a. Figure 8b shows that most of the sample images were annotated as being of low aesthetic quality.

**Figure 9:**
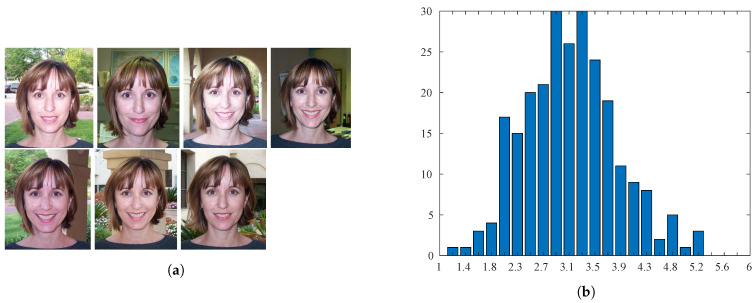
Sample images and distribution of scores for the Human Faces Score (HFS) database. (**a**) Samples are sorted by their aesthetic score (increasing from top left to bottom right). (**b**) Distribution of ground-truth scores.

The Human Faces Score (HFS) [22] database contains 250 photos of faces in the same pose with the same width of 240 pixels and a variable height. Specifically, seven images of 20 different people and 110 additional portrait images were collected. The face images of one subject are given in Figure 9a. The annotation of each image was obtained by having 25 human observers rate the image on a scale with values between 1 and 6 (the highest aesthetic quality), then calculating the Mean Opinion Score (MOS). In Figure 9b, the histogram of the MOSs for the database is shown.

**Figure 10:**
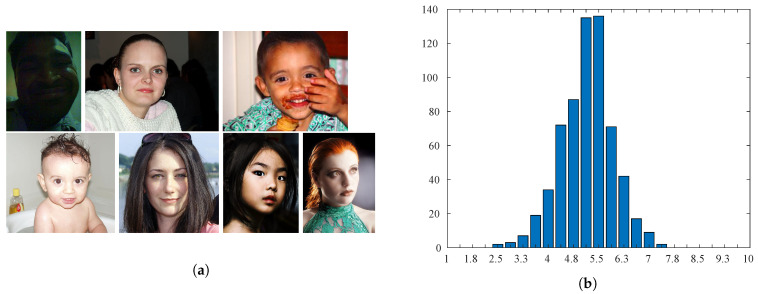
Sample images and distribution of scores for the Face Aesthetics Visual Analysis (FAVA) database. (**a**) Samples are sorted from by their aesthetic score (increasing from top left to bottom right). (**b**) Distribution of ground-truth scores.

The Face Aesthetics Visual Analysis (FAVA) database is a subset of the large-scale AVA dataset [28] containing various images with faces. The latter are portrayed in near-frontal positions. The smallest face in the database has a size of 198×212 pixels, while the largest has a size of 1462×1568 pixels. Each picture is associated with a value between 1 and 10 (the highest quality) corresponding to the average of around 210 collected individual scores (Figure 10b displays the histogram of the MOSs). Samples are shown in Figure 10a.

**Figure 11:**
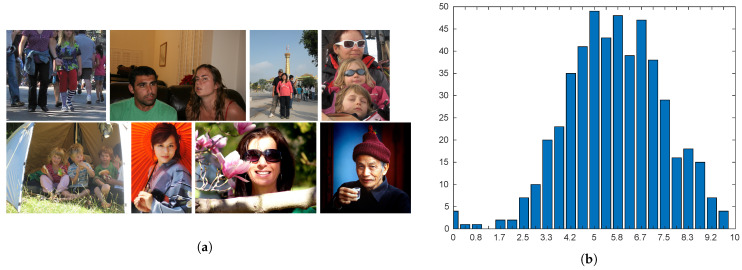
Sample images and distribution of scores for the Flickr database. (**a**) Samples are sorted by their aesthetic score (increasing from top left to bottom right). (**b**) Distribution of ground-truth scores.

The Flickr database was gathered from Flickr for general aesthetic assessment [1]. It consists of 500 images associated with a ground-truth score between 0 and 10, where 10 means high quality. Photos have the longest side corresponding to 1600 pixels and show a single face or a group of faces. The size of the smallest face in the database is 72×72 pixels, while the largest face almost completely covers the surface of the image with a size of 1462×1568 pixels. According to [19], only the biggest detected face is considered in each picture. Figure 11a shows samples from the database, while the distribution of the scores is reported in Figure 11b.

### 3.3. Experimental Setup

Binary aesthetic classification and aesthetic score regression were performed for each dataset presented previously.

For binary classification, the goal was to discriminate images into low-quality and high-quality aesthetics. To get the ground-truth for the databases that provide the MOSs (all except CUHKPQ, which already provides the low-/high-quality aesthetic labels), we followed the same protocol as in [19]. In this protocol, the datasets were first sorted by the Mean Opinion Score (MOS) values, then separated into two sets having the same number of samples to contain the images with the lowest and highest aesthetic scores, respectively.

In all the experiments, the GA was trained with a population of 100 individuals initialized by using the parameters (weights and bias) and their perturbed versions of a linear support vector machine (SVM) previously trained for aesthetic prediction. The learning parameters were empirically setup differently for classification and regression. More precisely, for classification, the number of generations was 200, the probability of crossover 80%, and the elitism (the percentage of individuals in the current generation who will survive for the next generation) 7%. For regression, the number of generation was 250, the crossover probability 85%, and finally the elitism 10%.

## 4. Results

In this section, we report the results achieved by our method on the four considered datasets separately in two different setups, then we compare our method’s performance with the ones of previous methods. Furthermore, we conduct a performance evaluation of the generalization ability of our method in a cross-database scenario. We ran all of our experiments on a desktop computer with an Intel Core i7-7700 CPU@3.60 GHz, 16 GB DDR4 RAM 2400 MHz, and NVIDIA Titan X Pascal with 3840 CUDA cores. The training time per experiment considering the 10 rounds of 10-fold cross-validation was 15 h on average. The inference time per image running the three CNN based feature extractors in parallel on the GPU was 0.08 s on average.

### 4.1. Performance on Single Databases

We performed two sets of experiments to evaluate how the context (background) influences the aesthetic judgment of images with faces. In the first set, the perceptual features were extracted from the entire image and the facial features from the face region only, as described in Section 2.2. In the second set, on the other hand, both the perceptual and facial features were extracted considering only the facial region. Additionally, we created a baseline exploiting a linear SVM instead of the GA for aesthetic quality estimation. This baseline highlights that the method benefits from the combination of features and the use of GA rather than a linear classifier. We employed a linear SVM for binary classification, while a linear SVR machine was used for continuous aesthetic score prediction. We report the performance obtained by considering a single feature vector at a time and then by all of their possible combinations.

#### 4.1.1. Experiments Considering the Whole Image

**Figure 12:**
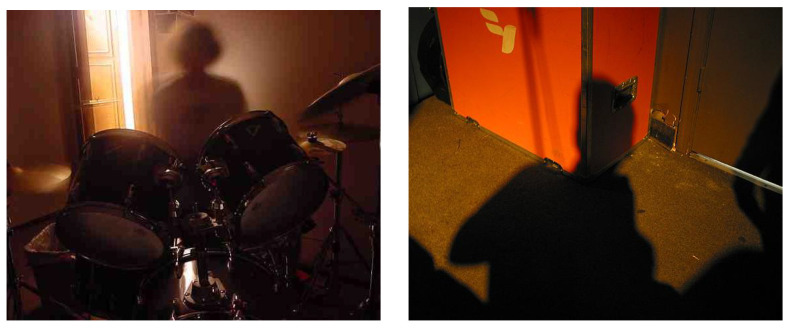
Low aesthetic quality samples from the CUHKPQ database in which the face is not present.

Table 1 reports the results for binary aesthetic classification in terms of GCR and F1-score. The best results for both metrics were achieved from the fusion of all the features. The performance on CUHKPQ was higher than that on the other two databases. This is because the CUHKPQ database is not very challenging. Although the images were taken from the “human” category, many of the low aesthetic quality images in the database have faces in random positions, which often do not look into the camera or are not present at all (see, for example, Figure 12).

**Figure 13:**
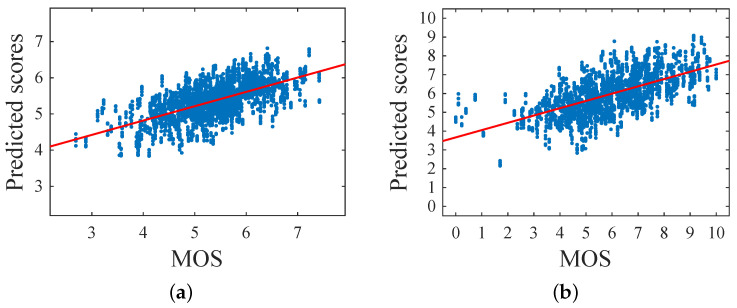
Scatter plots of predicted scores versus MOS for the databases (**a**) FAVA and (**b**) Flickr, using perceptual features extracted from the whole image.

**Figure 14:**
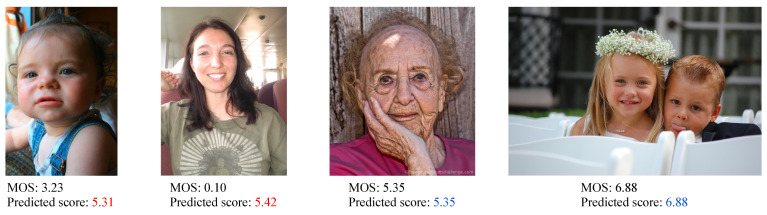
Images erroneously and correctly rated by our method GAwNorm-in-Norm trained on the whole image.

Table 2 depicts the results for the continuous aesthetic score in terms of the PLCC and the SROCC. The mixed-coded GA trained using norm-in-norm fitness outperformed all the other solutions for both metrics on FAVA and Flickr. In general, the GA based results were better than those obtained using SVR. Only the ranking based GA solution resulted in bad correlations probably because it needed more than 200 generations to converge to the optimum. Figure 13 shows the scatter plots of the predicted scores with respect to the MOS for both FAVA and Flickr in the 10 iterations. A linear regression function is drawn to highlight the silhouette of the fit. We can observe that both distributions were well fit. Figure 14 shows some examples of the predictions obtained by the GA optimized using norm-in-norm. The first two images were incorrectly evaluated; in fact, the predicted scores were higher than the MOS. The other two examples depict correctly rated images (MOS and predicted scores are equal). This may be due to the fact that the method does not penalize when face illumination is not homogeneous; instead, it is strongly influenced by positive facial expressions.

From the previous results, we can draw several conclusions. First, the combination of all the considered features achieved the best results for all the databases, and in most cases, the fusion of one of the perceptual features (quality or general aesthetics) with the facial features obtained better results than the combination of the perceptual features. Therefore, facial features are effective to model the aesthetics of images containing faces. Second, the performance results by the mixed-coded GA were close to those obtained by the SVM, but uses a lower feature set.

#### 4.1.2. Experiments Considering Only the Face Region

Results for binary aesthetic classification are reported in Table 3. As seen in the previous results, by fusing all the features, the best results were obtained. The performance for the FAVA dataset was higher than the one obtained by extracting features from the whole image. The reason might be that many images contain a small portion of background.

Performance results (in Table 4) for the continuous aesthetic score confirmed that the fusion of all the features was optimal and that the GA based solution obtained better results by using a smaller amount of features. In this set of experiments as well, ranking based GA performed worse than both smooth-L1 and norm-in-norm. The latter’s fitness allowed GA to achieve the best correlation for all the considered databases.

**Figure 15:**
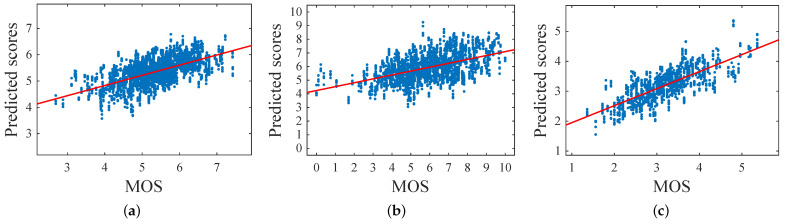
Scatter plots of predicted scores versus the MOS for the databases: (**a**) FAVA, (**b**) Flickr, (**c**), and HFS using perceptual features extracted from the face region.

Figure 15 depicts the scatter plots of predicted against MOS scores for FAVA, Flickr, and HFS. We used a linear regression function to highlight how the distributions were well fit.

The evaluation protocol we used (the same as [19]) for the HFS did not take into account whether images of the same subject were present in both training and testing; therefore, it was a person-dependent experiment. To assess the generalization ability of the proposed method, we performed a series of experiments in which we measured the performance of the best version of the proposed method (i.e., GAwNorm-in-Norm) by adopting person-independent cross-validation, where all the images of a subject must be in the training or the test set. In the latter experiments, the performance achieved for aesthetic classification degraded by 3% in terms of accuracy and by 0.04 in terms of PLCC between the MOS and the predicted scores.

#### 4.1.3. Comparison to Other Methods

A small number of methods have been developed and evaluated on the four databases considered. Furthermore, none of these methods has released the source code or executable program. Therefore, we compared our performance with that reported in the original paper only for the methods that adopted the same evaluation protocol used in this paper.

Baseline: The baseline is DeepIA, which was the method proposed by the authors for the aesthetic assessment of images with generic content.

Lienhard et al. [19]: Each face image was divided into four regions, namely the entire face, the face area, the eyes’ area, and the mouth area. These regions are described by 60 values (15 features in each of the four regions). Features correspond to sharpness, illumination, contrast, and color distribution measures. The late score fusion of the predicted scores from four classifiers was then performed to obtain the image aesthetic prediction.

Kairanbay et al. [20]: It consisted of a CNN trained using an augmentation scheme based on compositional photographic rules for low/high aesthetic quality classification of portrait images.

The previous methods were compared with our two best methods: the one proposed in [24], which we named GAwSmooth-L1, involving the use of GA optimized with smooth-L1, and its new version involving GA trained with norm-in-norm, which we named GAwNorm-in-Norm. Both methods exploit the combination of all the considered features extracted from the whole image.

Table 5 shows the comparison in terms of the GCR and PLCC. As is possible to see, on average, both GAwSmooth-L1 and GAwNorm-in-Norm improved the GCR by more than 3% with respect to the previous methods for binary aesthetic classification. GAwNorm-in-Norm outperformed the second method, which is our GAwSmooth-L1, by more than 2% on average in terms of PLCC.

### 4.2. Performance across Databases

In this section, we present the results of a set of experiments for evaluating the robustness and the generalization skills of our method in a cross-database scenario. In each case, one of the three regression databases was used for training, and the learned models were tested on the other two databases. We compared the SROCC obtained by our two methods GAwSmooth-L1 and GAwNorm-in-Norm. The results are reported in Table 6. It may be observed that the correlation on the test databases was not very high. This result could have been expected because the images of the databases are very different and probably also the criterion with which the ground truth was collected is not entirely consistent. The model trained on the Flickr database generalized better than the others. On the other hand, the model trained on FAVA did not estimate scores that correlated well with the MOS of the other testing databases; this was probably due to the fact that the MOS distribution of FAVA was very spiked on the average value of the MOS. Finally, GAwNorm-in-Norm was very effective for the aesthetic evaluation of faces; in fact, it generalized better than GAwSmooth-L1.

## 5. Conclusions

In this work, we propose a framework for the automatic estimation of the aesthetic quality of images containing faces. We exploit three different CNNs to encode global image aesthetics, perceptual quality, and facial attributes. A novel learning procedure based on mixed-coded genetic algorithms (GAs) is then applied for the combination of CNN features and image aesthetic prediction. We compare three different fitness functions for the optimization of the GA to predict the aesthetic score. Experiments on four benchmark datasets in both binary and continuous aesthetic score prediction tasks demonstrate the effectiveness of the proposed method. Furthermore, experimental results show that the fusion of perceptual features extracted from the entire image and facial features is more effective than modeling just the face region. The mixed-coded GA optimized using a recently proposed regression loss performs better than both using other fitness functions and using an SVM for aesthetics’ prediction. Finally, the performance evaluation in the cross-database setup is conducted to point out the robustness and generalization skills of our final method in comparison to other algorithms in the literature. Based on the experimental results, the robustness of the proposed method needs to be improved. To this end, we plan to extend our framework to include new features to characterize aspects of the image that are not taken into account at the moment, such as geometric composition and memorability, and let the genetic algorithm learn which features are relevant and which are not.

## Figures and Tables

**Table 1:** Results for the aesthetic quality categorization for each database by extracting perceptual features from the whole image. FA, AFFACT; GCR, good classification rate.

IQ	IA	FA	# of Features	GA	GCR (%)			F1-Score		
					CUHKPQ	FAVA	Flickr	CUHKPQ	FAVA	Flickr
✓			4096		93.2	63.6	64.3	0.86	0.63	0.63
	✓		4096		97.2	67.4	71.6	0.94	0.68	0.72
		✓	2048		97.0	70.0	66.2	0.94	0.70	0.66
✓		✓	6144		97.3	70.0	67.6	0.94	0.70	0.67
✓	✓		8192		97.4	67.0	73.3	0.95	0.68	0.73
	✓	✓	6144		98.2	71.2	73.6	0.96	0.71	0.73
✓	✓	✓	10,240		98.2	71.2	74.0	0.96	0.71	0.73
✓	✓	✓	8300	✓	97.5	70.7	73.9	0.95	0.71	0.73

**Table 2:** The Pearson linear correlation coefficient (PLCC) and the Spearman rank-order correlation coefficient (SROCC) of the aesthetic quality prediction for each database by extracting perceptual features from the whole image.

IQ	IA	FA	# of Features	GA	Fitness	PLCC		SROCC	
						FAVA	Flickr	FAVA	Flickr
✓			4096			0.38	0.36	0.38	0.37
	✓		4096			0.51	0.57	0.49	0.59
		✓	2048			0.55	0.48	0.53	0.47
✓		✓	6144			0.57	0.51	0.56	0.51
✓	✓		8192			0.36	0.56	0.51	0.58
	✓	✓	6144			0.62	0.62	0.60	0.63
✓	✓	✓	10,240			0.61	0.61	0.60	0.63
✓	✓	✓	10,229	✓	Smooth-L1	0.62	0.61	0.61	0.63
✓	✓	✓	10,233	✓	Norm-in-Norm	0.64	0.63	0.64	0.64
✓	✓	✓	10,242	✓	Ranking	0.58	0.60	0.60	0.61

**Table 3:** Results for the aesthetic quality categorization for each database by extracting perceptual features from the face region.

IQ	IA	FA	# of Features	GA	GCR (%)				F1-Score			
					CUHKPQ	HFS	FAVA	Flickr	CUHKPQ	HFS	FAVA	Flickr
✓			4096		92.0	72.4	63.3	59.1	0.84	0.71	0.63	0.60
	✓		4096		95.0	73.8	66.5	64.5	0.89	0.73	0.68	0.63
		✓	2048		97.0	71.0	70.0	66.2	0.94	0.72	0.69	0.65
✓		✓	6144		97.0	76.8	70.8	67.2	0.94	0.77	0.70	0.67
✓	✓		8192		95.4	75.1	65.6	65.0	0.90	0.74	0.67	0.64
	✓	✓	6144		97.1	78.0	71.7	65.4	0.94	0.78	0.72	0.64
✓	✓	✓	10,240		97.0	79.0	71.8	65.6	0.94	0.79	0.72	0.64
✓	✓	✓	8283	✓	96.1	79.0	71.1	66.5	0.92	0.79	0.71	0.64

**Table 4:** PLCC and SROCC of the aesthetic quality prediction for each database by extracting perceptual features from the face region.

IQ	IA	FA	# of Features	GA	Fitness	PLCC			SROCC		
						HFS	FAVA	Flickr	HFS	FAVA	Flickr
✓			4096			0.59	0.39	0.32	0.60	0.41	0.31
	✓		4096			0.66	0.50	0.48	0.66	0.49	0.47
		✓	2048			0.67	0.55	0.48	0.63	0.53	0.47
✓		✓	6144			0.71	0.56	0.49	0.70	0.56	0.48
✓	✓		8192			0.68	0.51	0.47	0.67	0.50	0.45
	✓	✓	6144			0.74	0.62	0.51	0.71	0.61	0.50
✓	✓	✓	10,240			0.74	0.61	0.51	0.73	0.60	0.50
✓	✓	✓	10,087	✓	Smooth-L1	0.76	0.61	0.51	0.74	0.60	0.49
✓	✓	✓	10,075	✓	Norm-in-Norm	0.80	0.62	0.52	0.75	0.62	0.51
✓	✓	✓	10,080	✓	Ranking	0.73	0.58	0.47	0.74	0.60	0.47

**Table 5:** Comparison with state-of-the-art methods for both aesthetic categorization and score prediction for all the considered databases. For CUHKPQ, only the binary ground-truth (low-/high- aesthetics) is provided; therefore, the PLCC cannot be estimated.

Methods	CUHKPQ	HFS		FAVA		Flickr	
	GCR (%)	GCR (%)	PLCC	GCR (%)	PLCC	GCR (%)	PLCC
Baseline	77.1	64.8	0.69	67.4	0.50	65.6	0.47
Lienhard et al. [19]	94.8	79.3	0.73	67.1	0.51	69.3	0.49
Kairanbay et al. [20]	–	–	–	65.3	–	–	–
Bianco et al. [24] (GAwSmooth-L1)	98.2	79.0	0.76	71.2	0.61	74.0	0.61
Proposed (GAwNorm-in-Norm)	98.2	79.0	0.80	71.2	0.64	74.0	0.63

**Table 6:** Cross-database performance in terms of SROCC. Each entire database was used for both training and testing.

Training	HFS		FAVA		Flickr	
Testing	FAVA	Flickr	HFS	Flickr	HFS	FAVA
Bianco et al. [24] (GAwSmooth-L1)	0.32	0.41	0.33	0.40	0.44	0.38
GAwNorm-in-Norm	0.37	0.45	0.36	0.42	0.46	0.41

## Data Availability

The data presented in this study are available in [1,15,22,28].
